# Developing symptom-based predictive models of endometriosis as a clinical screening tool: results from a multicenter study

**DOI:** 10.1016/j.fertnstert.2012.04.022

**Published:** 2012-09

**Authors:** Kelechi E. Nnoaham, Lone Hummelshoj, Stephen H. Kennedy, Crispin Jenkinson, Krina T. Zondervan, World Endometriosis Research Foundation Women's Health Symptom Survey Consortium

**Affiliations:** a Department of Public Health, University of Oxford, Oxford, United Kingdom; b World Endometriosis Research Foundation, London, United Kingdom; c Nuffield Department of Obstetrics & Gynaecology, University of Oxford, Oxford, United Kingdom; d Health Services Research Unit, University of Oxford, Oxford, United Kingdom; e Wellcome Trust Centre for Human Genetics, University of Oxford, Oxford, United Kingdom

**Keywords:** Endometriosis, predictive model, logistic regression

## Abstract

**Objective:**

To generate and validate symptom-based models to predict endometriosis among symptomatic women prior to undergoing their first laparoscopy.

**Design:**

Prospective, observational, two-phase study, in which women completed a 25-item questionnaire prior to surgery.

**Setting:**

Nineteen hospitals in 13 countries.

**Patient(s):**

Symptomatic women (n = 1,396) scheduled for laparoscopy without a previous surgical diagnosis of endometriosis.

**Intervention(s):**

None.

**Main Outcome Measure(s):**

Sensitivity and specificity of endometriosis diagnosis predicted by symptoms and patient characteristics from optimal models developed using multiple logistic regression analyses in one data set (phase I), and independently validated in a second data set (phase II) by receiver operating characteristic (ROC) curve analysis.

**Result(s):**

Three hundred sixty (46.7%) women in phase I and 364 (58.2%) in phase II were diagnosed with endometriosis at laparoscopy. Menstrual dyschezia (pain on opening bowels) and a history of benign ovarian cysts most strongly predicted both any and stage III and IV endometriosis in both phases. Prediction of any-stage endometriosis, although improved by ultrasound scan evidence of cyst/nodules, was relatively poor (area under the curve [AUC] = 68.3). Stage III and IV disease was predicted with good accuracy (AUC = 84.9, sensitivity of 82.3% and specificity 75.8% at an optimal cut-off of 0.24).

**Conclusion(s):**

Our symptom-based models predict any-stage endometriosis relatively poorly and stage III and IV disease with good accuracy. Predictive tools based on such models could help to prioritize women for surgical investigation in clinical practice and thus contribute to reducing time to diagnosis. We invite other researchers to validate the key models in additional populations.

**Discuss:** You can discuss this article with its authors and with other ASRM members at http://fertstertforum.com/nnoahamk-symptom-based-predictive-models-endometriosis/

Endometriosis is a chronic disease defined by the presence of endometrial-like tissue outside the uterus (1). Diagnostic delay ranging from 7 to 12 years is well documented in endometriosis (2–5) and contributes to the impaired quality of life and significant personal and societal costs associated with the condition (5). Sur-gery under general anaesthesia, most commonly a laparoscopy, is required to make a definitive diagnosis but this is expensive and potentially associated with complications (6, 7). The avail-ability of a noninvasive method to evaluate the likelihood of finding endometriosis at laparoscopy could reduce the diagnostic delay (8) and the number of women undergoing surgery unnecessarily. Accordingly, and in keeping with a consensus statement on research priorities in endometriosis (9), the last decade has seen much effort directed at identifying nonsurgical methods of diagnosing the disease or at least predicting its presence, which can then inform therapies for associated pelvic pain and/or infertility. Peripheral biomarkers (e.g., CA-125), endometrial biomarkers (e.g., endometrial nerve fibre density), and imaging have all been evaluated (10, 11), showing varying degrees of accuracy and potential clinical utility. Generally, however, these procedures predict endometriosis inadequately and are largely either invasive or semiinvasive themselves.

In recent years, the potential for using clinical information to predict endometriosis before surgery has been explored. In one such study of 90 women undergoing diagnostic laparoscopy (12), a classification tree based on symptoms, physical examination, and ultrasound findings correctly predicted only 38% of nonovarian endometriosis, an unsurprising finding given the general limited predictive accuracy of classification trees (13). Another related study described the development of an externally validated predictive model of pregnancy rates following a surgical diagnosis of endometriosis, but prediction was not based solely on symptoms. Two other key studies have evaluated the likelihood of finding deep infiltrating (14) and bladder (15) endometriosis based on standardized symptom questionnaires, showing “acceptable” and “excellent” diagnostic value, respectively, for these subtypes. However, the studies used relatively small samples and, although internal cross-validation within the data set was attempted in one of the studies (14) through a bootstrap method, the models have never been validated in external data sets. Because predictive models always perform better on data on which they were generated than on new data, external validation is essential before implementing predictive models in clinical practice (16–18).

In addition to the limitations highlighted above, the global utility of predicting endometriosis clinically is often hampered by inconsistencies in the definition of the disease and associated symptoms across studies and populations (19). The question as to whether a valid predictive model based on symptoms associated with endometriosis, generalizable across populations in different countries, can be generated, led us to initiate the Women's Health Symptom Survey (WHSS). Its aims were to [1] develop symptom-based models that predict the likelihood of finding endometriosis at laparoscopy in women who are being investigated for endometriosis-associated pain and/or infertility and [2] determine the sensitivity and specificity of the models in predicting the likelihood of a diagnosis of endometriosis in a separate validation sample of women. Although physical examination plays an important role in the clinical evaluation of symptomatic women, in this study, the models were generated solely on the basis of symptom/medical history profiles and ultrasound evidence, to allow for standardized evaluation across centers and countries. Such models, if shown to have good predictive power, could be used to generate a screening assessment tool to prioritize women for further surgical evaluation.

## Materials and methods

### Study Population

The WHSS was a two-phase (model development/validation), clinic-based study in 19 hospitals in 13 countries. Between September 2008 and January 2010, we prospectively recruited 1,396 consecutive pre-menopausal women, aged 18–45, undergoing diagnostic laparoscopy because of at least one of the following symptoms: dysmenorrhoea (34.0% in phase I vs. 31.8% in phase II), dyspareunia (12.3% in phase I vs. 14.5% in phase II), nonmenstrual pelvic pain (36.1% in phase I vs. 37.0% in phase II), menstrual dyschezia (6.9% in phase I vs. 8.0% in phase II), or infertility (56.5% in phase I vs. 47.5% in phase II). Women with a previous surgical diagnosis of endometriosis, amenorrhoea or current pregnancy, or who had taken hormonal medication (including combined oral contraception) within the previous 3 months, were excluded.

During the model generating phase I (September 2008 to June 2009), consenting women who met the inclusion criteria completed, prior to their scheduled surgery, a 25-item self-administered questionnaire in their own language (www.endometriosisfoundation.org/WERF-WHSS-Questionnaire-English.pdf). During the model validating phase II (July 2009 to January 2010), premenopausal women, aged 18–45 years, were recruited using the same inclusion and exclusion criteria as in phase I; they completed the same 25-item questionnaire before their surgery. The questionnaire incorporated items to elicit women's past medical, obstetric, and family histories, as well as items to evaluate the intensity and frequency of pelvic pain. Pelvic pain intensity was assessed on 11-point numerical pain rating scales (20) ranging in possible values from 0 (no pain) to 10 (worst possible pain). The questionnaire also included standardized questions previously validated in women with pelvic pain or other symptom groups. These instruments included [1] the IBS Rome III questionnaire to identify women with pelvic pain symptoms due to irritable bowel syndrome (21) and [2] standardized pelvic pain symptom assessment used in earlier studies in Oxford (22, 23). The questionnaire also asked for sociodemographic, lifestyle, and physical attributes. Experienced gynecol-ogists recorded the laparoscopic findings in a standard manner (http://www.endometriosisfoundation.org/WERF-GSWH-WHSS-surgical-sheet.pdf). For those women who had preoperative pelvic ultrasound (84.7% and 92.2% of women in phases I and II, respectively), imaging findings were recorded on the surgical sheet.

Cases were defined as women in the study populations who, at laparoscopy, were found to have endometriosis—diagnosed on visual evidence alone according to the European Society of Human Reproduction and Embryology guideline (1) and staged using the revised American Fertility Society classification: I (minimal), II (mild), III (moderate), or IV (severe) (24). Controls were women in the study populations without endometriosis (with or without other diagnoses) at laparoscopy. The Mid- and South Buckinghamshire Research Ethics Committee in the United Kingdom approved the study, followed by approval from all the local ethics committees.

### Statistical Analysis

#### Model generation

Women who had [1] any stage of endometriosis and [2] stage III or IV endometriosis were compared to controls. To compare cases and controls on categorical variables, Pearson's χ^2^ tests or the Fisher's exact test were used where appropriate. Continuous parametric variables were assessed using the Student's *t*-test and nonparametric variables with the Mann-Whitney *U* test.

**Figure 1:**
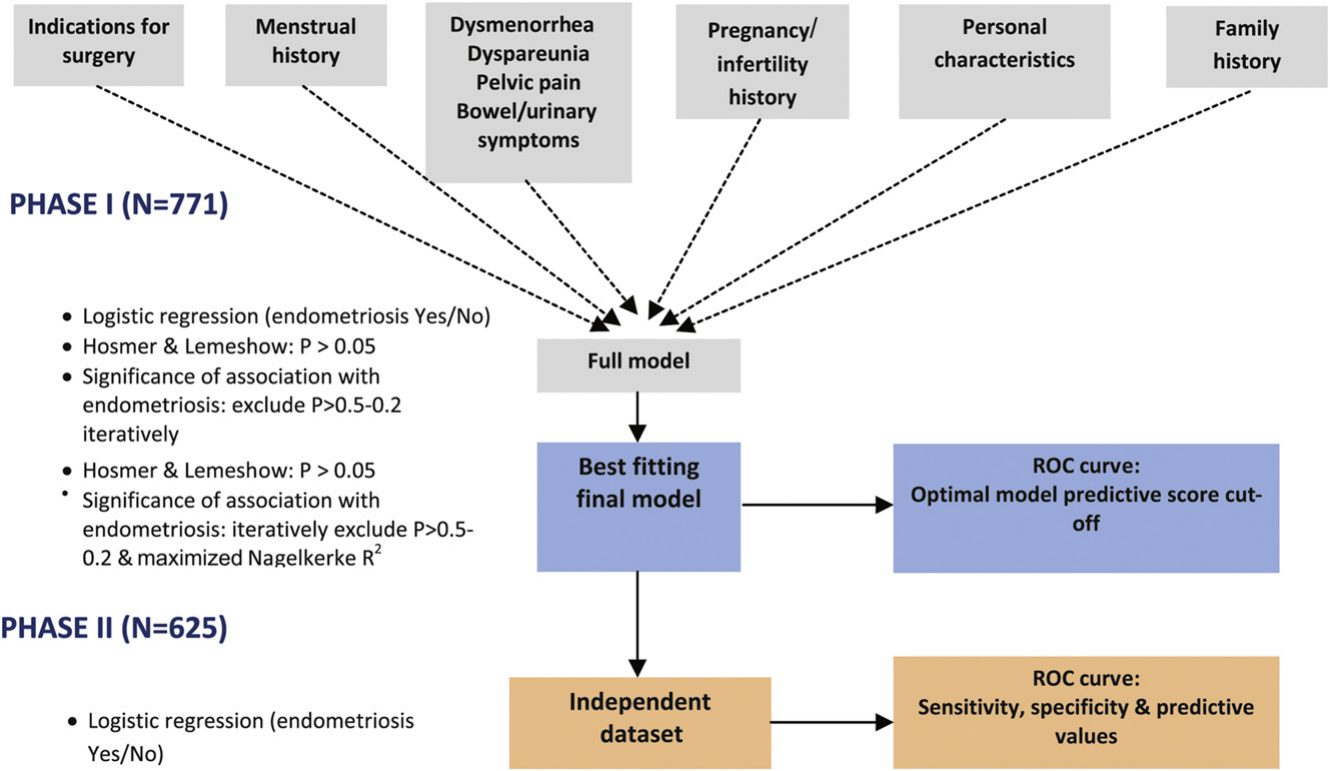
Model building and validation approach in the WHSS.

As the outcome of interest (presence of any-stage and stage III or IV endometriosis) was binary in nature, logistic regression was used for the predictive modeling. The WHSS questionnaire contained more than 200 variables, which, if entered into one logistic regression model, would have resulted in over fitting of models to the data. As a guide, in the final model, the number of degrees of freedom (df) should not exceed approximately 10% of the number of observations in the smaller outcome category (25). We therefore employed a tiered approach to building the predictive model in phase I (Fig. 1).

First, groups of clinically related variables were assessed in a multivariate logistic regression framework, to assess which variables within each group showed little or no association with endometriosis, and could therefore be excluded. For each group, this was done iteratively by first excluding variables for which tests of association with endometriosis resulted in significance levels of *P*>.5, progressing to dropping variables with *P*>.2, resulting in a submodel for each of the groups. In each group, the goodness of fit of the submodel to the data was tested using the Hosmer and Lemeshow test (26), whereas the drop in Nagelkerke's *R*^2^ (assessing the disease variance explained by the variables in the model) when removing variables was not to exceed 10%. Variables from each of the submodels were then included in the complete models, which were again reduced iteratively by considering [1] the Hosmer and Lemeshow test for goodness of fit, [2] the drop in Nagelkerke's *R*^2^ when removing variables, and [3] the significance of the association of each variable in the model with endometriosis (dropping variables from *P*>.5 down to *P*>.2). This resulted in best-fitting final models for the prediction of any-stage and stage III and IV endometriosis based on the phase I data. For each of the two diagnostic outcomes, a model including and excluding preoperative ultrasound evidence of cysts/nodules was generated (Supplemental Table 1). Final models were subsequently fitted to phase II data to assess their predictive performance (see model validation below). In addition, reduced models were generated, which excluded variables in the best-fitting final models that had opposite directions of effect in phase I and II data sets. These reduced models were also assessed for performance; thus, a total of eight model types were generated and assessed (Supplemental Table 1).

#### Model validation

To illustrate the performance of the derived predictive models in the phase I population data, and the relative drop in performance when externally validating in phase II data, the accuracy of the models in predicting any-stage and stage III and IV endometriosis was assessed by analyzing the receiver operating characteristic (ROC) curve in both phase I and II. The ROC curve displays the relationship between sensitivity and 1-specificity and the area under the ROC curve (AUC) depicts how well the model distinguishes women with and without endometriosis; a model with a greater AUC has a better-performing risk function. Model sensitivity, specificity, and positive and negative likelihood ratios were also calculated, and the best model cut-off points were considered to be those that corresponded to the highest sum of specificity and sensitivity.

All univariate and logistic regression analyses were done using the Statistical Package for the Social Sciences 16.0 (SPSS, Inc.); prediction analyses in both phase I and II populations were conducted within the binary logistic module in SPLUS 6.0 (TIBCO Software, Inc.); and ROC analysis using MedCalc 11.6 (MedCalc Software).

## Results

### Description of the Predictive Models

**Supplemental Figure 1:**
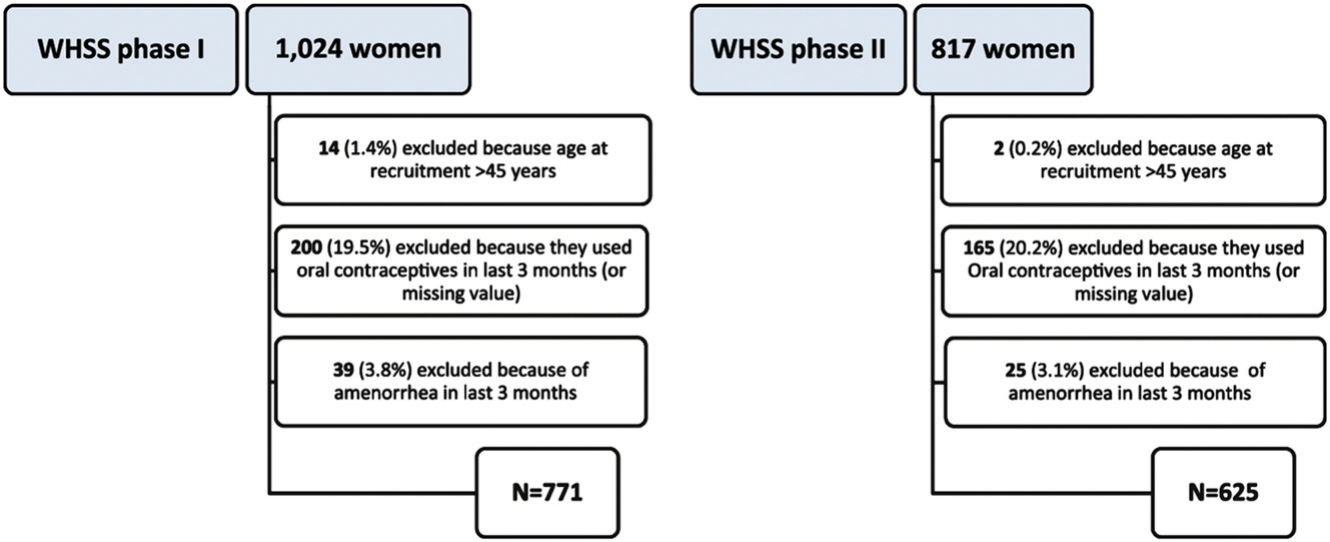
Recruitment flowchart for WHSS phases I and II.

As shown in Supplemental Figure 1, 771 (phase I) and 625 (phase II) of the women recruited met the inclusion criteria and had complete surgical information at the close of the study. Among participants, the proportions of cases at centers varied from 35% in Ibadan to 97% in Guangzhou. Supplemental Table 2 shows the average age of case and control women in both phases and the frequency of pathology found at surgery. Endometriosis was diagnosed in 360 (46.7%) women in phase I and 364 (58.2%) women in phase II.

The results of the best-fitting final models for any-stage and stage III and IV endometriosis, respectively, are shown in Supplemental Table 3 and Table 1, respectively. The results of the reduced models (only retaining variables with consistent evidence in phases I and II [see Methods]) are not shown, but these variables are highlighted in bold. The full “any-stage no ultrasound” model 1 (see Supplemental Table 3) had 26 variables but 12 of these (46%) were dropped in the reduced model 2 (see Methods). Similarly, 9 of 23 variables (39%) in the full “any-stage ultrasound” model 3 were dropped in the corresponding reduced model 4. Notably, menstrual dyschezia (pain on opening bowels during periods) and a medical history of benign ovarian cysts were most strongly associated with any-stage endometriosis in models with ultrasound (Phase II OR = 3.12, 95% CI = 1.07–9.10, *P*=.037, and OR = 2.92, 95% CI = 1.35–6.30, *P*=.006, respectively) and without ultrasound (Phase II OR = 3.47, 95% CI = 1.40–8.57, *P*=.007, and OR = 4.15, 95% CI = 2.19–7.86, *P*<.001, respectively). Rectal bleeding during menstruation, IBS (Rome III), unspecified functional bowel disorder, duration of smoking, subfertility due to blocked tubes, and ethnicity (Asian/Oriental and other/mixed) were inconsistently associated with endometriosis in both any-stage endometriosis models. The any-stage ultrasound model 3 explained substantially more variability in endometriosis than the any-stage no ultrasound model 1 (Nagelkerke's *R*^2^ = 0.54 vs. 0.44 in phase I). Although the any-stage model including ultrasound (model 3) retained its *R*^2^ value of 0.54 in phase II, its value dropped to 0.30 for model 1, indicating a better predictive performance of models, including ultrasound evidence.

The full stage III and IV no ultrasound model 5 (Table 1), had 23 variables, but 6 of these were dropped in the reduced model 6. In comparison, 3 of 18 variables in the full stage III and IV ultrasound model 7 were dropped in the corresponding reduced model 8. Menstrual dyschezia, a medical history of benign ovarian cysts, and Black ethnicity were most strongly associated with stage III and IV endometriosis, whereas other/mixed ethnicity was the only variable inconsistently associated with endometriosis in both any-stage endometriosis models in phases I and II. Although model 7 including ultrasound explained greater variability in diagnosis of stage III and IV endometriosis than model 5 without ultrasound (Nagelkerke's *R*^2^ = 0.57 vs. 0.47 in phase I), the drop in *R*^2^ was comparable for both models (to *R*^2^ = 0.51 vs. 0.42). All four full predictive models fitted the data well as shown by the Hosmer and Lemeshow test *P* values (all *P*>.05).

### Validation of the Predictive Models

**Figure 2:**
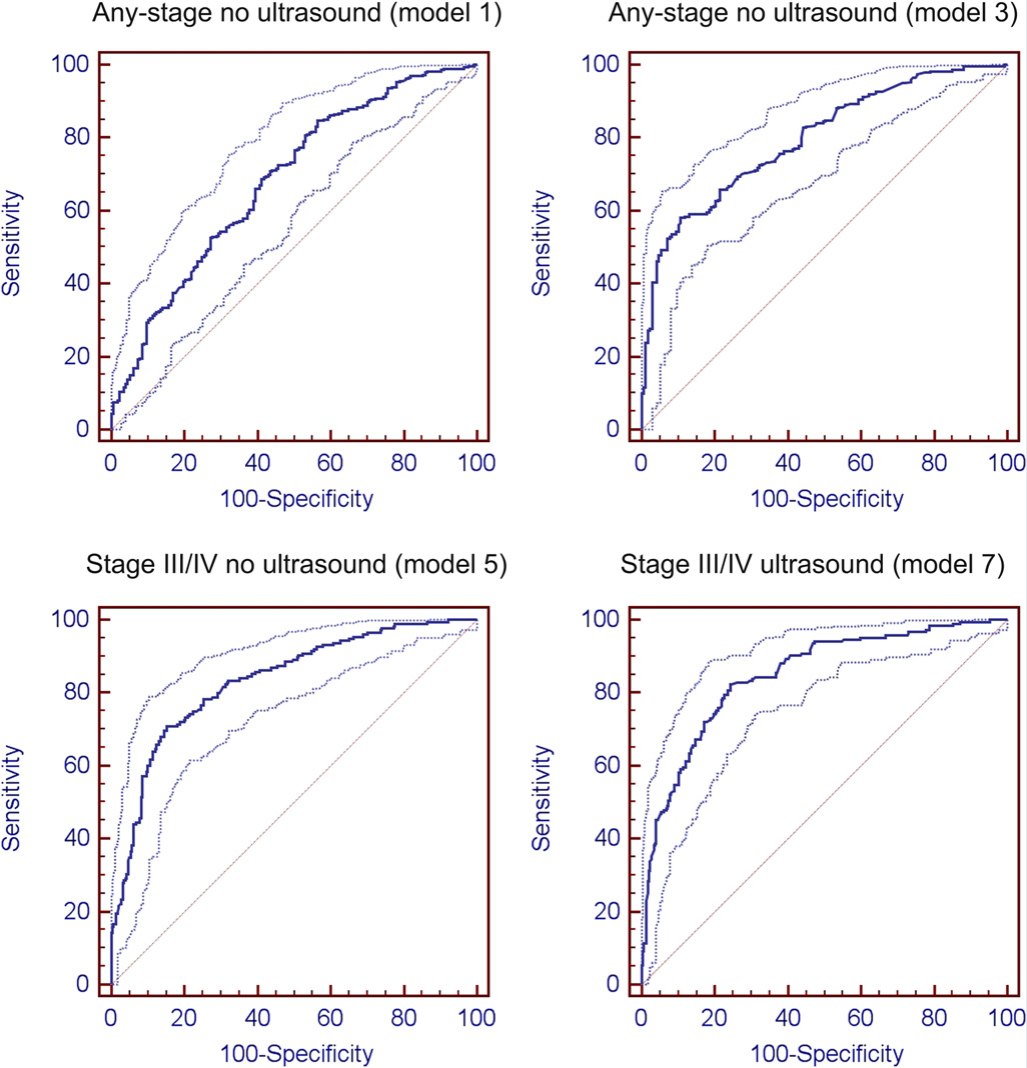
Receiver operating characteristic curves for the full models predicting any-stage and stage III or IV endometriosis.

All four full models were evaluated for their predictive performance in the phase II data. As expected, ultrasound evidence alone showed high sensitivity but very low specificity in the prediction of any or stage III or IV endometriosis (Table 2), that is, positive ultrasound evidence was a good predictor of the presence of endometriosis, but negative evidence was a very poor predictor of absence of disease. As shown in Table 2 and Figure 2, the full any-stage no ultrasound model 1 had good discrimination in phase I data (AUC = 84.2, 95% CI = 81.1–87.0, *P*<.0001), but its performance was reduced substantially when applied to the phase II validation data set (AUC = 68.3, 95% CI = 63.9–72.4, *P*<.0001). Predictive ability in phase II data was somewhat improved in the reduced model 2 to AUC = 72.2 (95% CI = 68.1–76.1, *P*<.001), although the optimal model cut-off of 0.59 would provide a low sensitivity of 54%.

Similarly, the full any-stage ultrasound model 3 had good discrimination in phase I data (AUC = 87.3, 95% CI = 84.2–90.0, *P*<.0001), and although some reduction in its performance was evident when applied to phase II data (AUC = 80.0, 95% CI = 75.6–83.3, *P*<.0001), this reduction was not as substantial as for the any-stage no ultrasound model 1. The corresponding reduced model 4 improved predictive ability in phase II data to AUC = 85.1 (95% CI = 81.5–88.2, *P*<.001), its optimal model cut-off of 0.51 providing a sensitivity of 80% and a specificity of 77% (cf. respective values of 0.80, 58% and 89% for the full model).

The full stage III or IV no ultrasound model 5 had good discrimination in phase I data (AUC = 87.3, 95% CI = 84.3–89.8, *P*<.0001) and retained good predictive power when applied to the phase II data set (AUC = 83.3, 95% CI = 79.6–86.6, *P*<.0001). Notably, the reduced model 6 did not offer improvement in model performance (AUC = 81.1, 95% CI = 77.2–84.6, *P*<.0001). The reduced model optimal cut-off was 0.24, with a sensitivity of 74% and specificity of 78% (cf. respective values of 0.45, 71%, and 85% for the full model).

Similarly, the stage III or IV ultrasound model 7 had good discrimination in both phase I (AUC = 90.8, 95% CI = 88.1–93.0, *P*<.0001) and phase II data (AUC = 84.9, 95% CI = 081.4–88.0, *P*<.0001). The corresponding reduced model 8 did not improve model performance in phase II data (AUC = 85.5, 95% CI = 82.0–88.5, *P*<.0001). The reduced model optimal cut-off was 0.29, with a sensitivity of 80% and a specificity of 80% (cf. respective values of 0.24, 82%, and 76% for the full model).

## Discussion

In this multicenter study of symptomatic premenopausal women presenting with symptoms that were potentially indicative of endometriosis, we show that a combination of symptom characteristics and variables in the medical history, with or without ultrasound evidence of cysts/nodules, can predict the finding of stage III and IV endometriosis at laparoscopy with reasonably good accuracy. The best-fitting predictive model included, along with ultrasound evidence, menstrual dyschezia, ethnicity, and a history of benign ovarian cysts as the variables with the strongest predictive performance. These variables are mostly disease risk factors reported in previous studies. Specifically, menstrual dyschezia is strongly associated with deep infiltrating endometriosis, a severe form of the disease (27), which had a relatively low prevalence in our clinical population (7.0% in phase I, and 7.8% in phase II). The positive association of stage III and IV endometriosis with Black ethnicity, however, conflicts with previous reports which suggest that White and Asian women have a greater risk of disease (28), although these reports generally relate to any-stage, rather than stage III and IV, endometriosis.

In this study, we report the development in one sample population, and validation in another drawn from the same source population, of models predicting [1] any-stage and [2] stage III and IV, endometriosis, with or without ultrasound scan evidence. The ability to predict any-stage endometriosis in the model excluding ultrasound evidence was generally poor (AUC = 68.3). Some improvement (AUC = 72.2) could be gained by removing from the model variables with inconsistent association with endometriosis across phase I and II data, but the external validity of the reduced model would need to be evaluated in an independent data set. When including ultrasound scan evidence, the prediction of any-stage endometriosis was improved (AUC = 80.0), but the optimal model cut-off results in a relatively low sensitivity (58%), which reduces the utility of the model as a potential clinical screening tool. The poor predictability of any-stage endometriosis is not surprising given that similar findings are reported for models based on serum markers (29), and stage I (minimal) endometriosis is considered pathogenetically to be different to stage III and IV disease (30–33). In contrast to our findings, both any-stage and stage III and IV endometriosis were reported to be predictable from the medical history of 1,079 prospectively recruited subfertile women in Portugal (34). However, as the predictive models were not validated in an independent data set, their findings should be interpreted with some caution.

The models predicting stage III and IV endometriosis (±ultrasound evidence) showed much better performance. However, the model that included ultrasound evidence showed better performance, which is not surprising given the value of ultrasound to diagnose ovarian endometriomas (35)and deep infiltrating endometriosis affecting the bowel (36). Optimal model cut-offs resulted in sensitivities of 70.9% and 82.3%, and specificities of 84.7% and 75.8%, for models excluding and including ultrasound evidence, respectively. In contrast to any-stage models, only marginal improvement could potentially be gained by excluding variables with inconsistent association with stage III and IV endometriosis across the data from phases I and II; this relative consistency in association across phases provided further evidence of the superior predictability of stage III and IV endometriosis over “any stage” disease.

The stage III and IV endometriosis models could, in addition to ultrasound and physical examination, be used to prioritize women presenting with symptoms for laparoscopy in clinical practice, that is, mirroring the setting in which the present study was conducted, or to initiate medical therapies sometimes reserved until a surgical diagnosis of endometriosis has been made. To what extent the models have predictive power in other settings (e.g., self-selected women with pelvic pain symptoms in the general population) is unknown, and therefore the utility of the tool should not be advocated for this purpose. Indeed, although identifying a noninvasive diagnostic test for endometriosis is an explicit priority in endometriosis research, other authors have cautioned that such a tool could be misappropriated as a population screening tool for a disease that may not fit a population screening model (37).

A potential argument against the use of the models to prioritize symptomatic women for surgery is that a high prevalence of other pathologies was found among controls in this study (72%), which could warrant surgical intervention. However, whether surgical intervention would be deemed appropriate for these pathologies, or indeed whether they were likely to be the underlying reason for the symptoms or a coincidental finding, is a matter for debate. We believe that the stage III and IV prediction models in particular are potentially useful clinically, as the likely presence of moderate/severe disease would be a good basis for prioritization of surgical exploration and intervention.

As far as we know, this is the first study to use robust modeling techniques for model generation, followed by external validation to generate and validate symptom-based predictive models of endometriosis in a large prospectively recruited cohort of women across different countries and ethnicities. Previous attempts, focused on subtypes of endometriosis (13, 14), have been hampered by small sample sizes (11), and failed to validate models in populations independent to those from which model parameters were generated (34). The enrollment of women from diverse backgrounds according to a uniform set of criteria potentially addresses issues with the global utility of clinical prediction of endometriosis arising from inconsistencies in disease definition across studies and population. We invite other research groups to validate the key models in this paper in additional populations, as well as in subgroups that may be of specific clinical or population-based interest (e.g., those women who had infertility as the only surgical indication, who had biopsy-proven disease, or who were of a particular ethnicity).

Although the WHSS was designed to improve on the limitations of earlier studies, it had itself potential limitations. First, endometriosis was diagnosed visually, without histologic confirmation, although this followed the European Society of Human Reproduction and Embryology guideline (23), based on the premise that negative histology does not exclude the presence of disease. Consequently, disease status may have been inappropriately assigned; however, participating hospitals were experienced in diagnosing endometriosis. In a separate diagnostic validation study, 29 surgeons from the participating centers viewed nine standardized videos to allow, in a blinded manner, the assessment of consistency in diagnosis and staging of disease. Preliminary analysis suggested substantial inter-rater agreement in disease identification and staging (both Fleiss *k* > 0.60; C. Becker and K. May, unpublished data). Second, the generation of the models with reduced numbers of variables was based on both phase I and phase II data. More parsimonious models are always preferable (38); however, because they are partly based on phase II evidence, they would require additional external validation. Third, although a strength of the study was that results were generated using data from a wide variety of clinical centers worldwide covering a range of patient profiles, this meant that the results may have been affected to some extent by selection bias possibly arising from [1] differential frequency of concomitant pathologies, in particular the higher proportion of women with nonendometriotic adhesions amongst controls compared to cases (32.4% vs. 14.7% in phase I), and [2] the significant variations across centers in proportions of cases in the sample populations. This is another reason why we call for further independent validation of the models in additional clinical populations, which are likely to each have their own unique patient population.

In conclusion, the diagnostic delay, high investigation costs, and personal suffering associated with endometriosis might be reduced by access to a screening tool that predicts endometriosis with good accuracy in women presenting in a clinical setting. Although prediction of any-stage endometriosis is relatively poor, the symptom-based models developed and validated in this study predict stage III and IV endometriosis with a good degree of accuracy. They suggest that such a tool might help to prioritize women for surgical investigation in gynecologic practice.

## Figures and Tables

**Supplemental Table 1:** Model types generated based on phase I data alone (“full” models) and on additional association information from phase II data (“reduced” models).

Diagnosis	Model name	
	Full model	Reduced model
Any-stage endometriosis	Any-stage no ultrasound (model 1)	Any-stage no ultrasound (model 2)
	Any-stage ultrasound (model 3)	Any-stage ultrasound (model 4)
Stage III or IV endometriosis	Stage III and IV no ultrasound (model 5)	Stage III and IV no ultrasound (model 6)
	Stage III and IV ultrasound (model 7)	Stage III and IV ultrasound (model 8)

**Supplemental Table 2:** Characteristics of the 1,396 women in the Women's Health Symptom Survey, phases I and II.

	Phase I (N = 771)		Phase II (N = 625)	
	Cases (n = 360)	Controls (n = 411)	Cases (n = 364)	Controls (n = 261)
Age (y), mean ± SD	31.2 ± 6.0	31.0 ± 6.7	32.4 ± 6.3	32.2 ± 6.2
Ultrasound scan	312 (86.7)	340 (82.7)	341 (93.7)	235 (90.0)
Suggested endometrioma	108 (34.6)	12 (3.5)	165 (48.4)	6 (2.6)
Suggested nodule	17 (5.4)	1 (0.3)	35 (10.3)	11 (4.7)
Pathology observed at surgery	360 (100.0)	298 (72.5)	364 (100.0)	188 (72.0)
Any-stage endometriosis	360 (100.0)	0 (0.0)	364 (100.0)	0 (0.0)
ASRM stage				
I	118 (32.8)	0 (0.0)	91 (25.0)	0 (0.0)
II	59 (16.4)	0 (0.0)	39 (10.7)	0 (0.0)
III	89 (24.7)	0 (0.0)	101 (27.7)	0 (0.0)
IV	91 (25.3)	0 (0.0)	128 (35.2)	0 (0.0)
Bladder nodule	16 (4.4)	0 (0.0)	12 (3.3)	0 (0.0)
Cul-de-sac nodule	46 (12.8)	0 (0.0)	61 (16.8)	0 (0.0)
Bowel nodule	25 (6.9)	0 (0.0)	18 (4.9)	0 (0.0)
Fibroids	58 (16.1)	62 (15.1)	77 (21.2)	57 (21.8)
Adhesions	53 (14.7)	133 (32.4)	77 (21.2)	65 (24.9)
Nonendometriotic ovarian cyst	44 (12.2)	91 (22.1)	39 (10.7)	71 (27.2)

*Note:* Values are n (%) unless noted otherwise.

**Supplemental Table 3:** Association results and regression coefficients from best-fitting final full models for any-stage endometriosis excluding (model 1) and including (model 3) ultrasound evidence in phase I, and when subsequently applied to phase II.

Model	Variable	Phase I			Phase II		
		OR (95% CI)	*P* value	β	OR (95% CI)	*P* value	β
Any-stage no ultrasound (model 1)	Surgery/consultation for painful periods∗	1.89 (1.15–3.10)	.012	0.635	1.97 (1.12–3.47)	.019	0.679
	Surgery/consultation for ovarian cyst∗	1.63 (0.90–2.95)	.11	0.488	1.53 (0.84–2.80)	.17	0.427
	Typical duration of periods (days)^a^	0.84 (0.73–0.96)	.010	−0.177	1.03 (0.89–1.19)	.73	0.026
	Menstrual flow^b^	1.24 (0.85–1.80)	.26	0.214	0.93 (0.63–1.37)	.70	−0.076
	Pelvic pain during periods∗	3.70 (2.22–6.15)	<.001	1.307	1.12 (0.67–1.88)	.67	0.112
	Pain on opening bowels during periods∗	3.85 (1.88–7.91)	<.001	1.349	3.47 (1.40–8.57)	.007	1.24
	Bleeding on opening bowels during periods	0.11 (0.02–0.63)	.013	−2.206	1.88 (0.11–31.1)	.66	0.632
	Blood in urine during periods∗	0.20 (0.03–1.36)	.10	−1.604	0.62 (0.11–3.42)	.59	−0.471
	Pelvic pain other times than periods or intercourse∗	2.47 (0.67–9.09)	.18	0.902	1.22 (0.29–5.20)	.78	0.202
	Time since pelvic pain first started^c^	0.84 (0.62–1.14)	.26	−0.175	1.06 (0.76–1.48)	.74	0.056
	Worst severity of pelvic pain at last pelvic exam∗^d^	1.20 (1.05–1.37)	.006	0.184	1.14 (0.99–1.33)	.074	0.134
	Incomplete emptying of bladder in general∗	0.80 (0.65–0.99)	.036	−0.221	0.89 (0.71–1.13)	.35	−0.112
	Pelvic pain after urination in general∗	0.76 (0.54–1.08)	.12	−0.271	0.54 (0.36–0.82)	.004	−0.618
	IBS-U (Rome III): unspecified functional bowel disorder	0.19 (0.04–0.85)	.029	−1.657	3.40 (0.25–47.0)	.36	1.22
	Number of live births∗^e^	0.57 (0.42–0.76)	<.001	−0.571	0.61 (0.44–0.83)	.002	−0.501
	Number of moles∗^f^	1.32 (0.99–1.75)	.058	0.276	1.23 (0.90–1.68)	.19	0.209
	Ever diagnosed blocked tubes as reason for subfertility	0.32 (0.17–0.58)	<.001	−1.149	0.99 (0.54–1.82)	.98	−0.009
	Ovarian cysts (benign) ever diagnosed∗	2.19 (1.21–3.96)	.009	0.784	4.15 (2.19–7.86)	<.001	1.42
	Polycystic ovarian syndrome ever diagnosed	0.33 (0.15–0.75)	.008	−1.100	1.20 (0.45–3.23)	.72	0.182
	Scoliosis ever diagnosed	3.38 (1.02–11.1)	.045	1.218	0.80 (0.27–2.36)	.69	−0220
	Family history of endometriosis in first- or second-degree relative∗	2.92 (0.99–8.61)	.052	1.072	1.77 (0.67–4.64)	.25	0.568
	Family history of asthma in first- or second-degree relative	0.42 (0.22–0.80)	.009	−0.863	1.31 (0.61–2.86)	.49	0.273
	Asian or Oriental ethnicity^g^	0.90 (0.47–1.72)	.74	−0.108	1.37 (0.65–2.87)	.41	0.314
	Black ethnicity∗^g^	2.02 (0.77–5.29)	.15	0.705	5.70 (2.53–12.8)	<.001	1.74
	Other/mixed ethnicity^g^	0.70 (0.36–1.36)	.30	−0.353	1.31 (0.60–2.84)	.50	0.267
	Duration of smoking (years)^a^	0.96 (0.93–0.99)	.005	−0.043	1.02 (0.98–1.06)	.42	0.018
	Phase I: Nagelkerke's *R*^2^ = 0.44; Hosmer-Lemeshow test = 0.76. Phase II: Nagelkerke's *R*^2^ = 0.30; Hosmer-Lemeshow test = 0.22						
Any-stage ultrasound (model 3)	Ultrasound evidence of cyst/nodule∗	18.9 (7.5–47.3)	<.001	2.939	122 (27–548)	<.001	4.806
	Surgery/consultation for painful periods∗	4.37 (2.28–8.36)	<.001	1.474	1.65 (0.80–3.44)	.18	0.503
	Surgery/consultation for infertility	0.65 (0.36–1.19)	.17	−0.428	1.40 (0.72–2.74)	.32	0.338
	Surgery/consultation for heavy periods	0.30 (0.13–0.70)	.006	−1.202	1.18 (0.51–2.75)	.70	0.165
	Pain on opening bowels during periods∗	5.33 (2.18–13.0)	<.001	1.637	3.12 (1.07–9.10)	.037	1.137
	Bleeding on opening bowels during periods	0.01 (0.0–0.08)	<.001	−4.753	2.81 (0.14–58.0)	.50	1.035
	Pelvic pain other times than periods or intercourse∗	7.23 (2.18–24.4)	.001	1.978	1.16 (0.33–4.09)	.82	0.144
	Average severity of pelvic pain∗^d^	0.79 (0.64–0.98)	.031	−0.237	0.82 (0.64–1.06)	.13	−0.196
	Worst severity of pelvic pain at last pelvic exam∗^d^	1.25 (1.06–1.48)	.010	0.224	1.22 (0.98–1.53)	.081	0.199
	Incomplete emptying of bladder in general∗	0.62 (0.47–0.80)	<.001	−0.485	0.84 (0.63–1.13)	.25	−0.174
	IBS-U (Rome III): unspecified functional bowel disorder	0.09 (0.01–0.60)	.013	−2.433	1.86 (0.11–31.5)	.67	0.621
	Number of live births∗^e^	0.71 (0.50–1.00)	.051	−0.346	0.60 (0.40–0.91)	.016	−0.507
	Number of moles^f^	1.26 (0.90–1.78)	.18	0.232	0.98 (0.67–1.42)	.91	−0.021
	Ever diagnosed blocked tubes as reason for subfertility	0.37 (0.18–0.78)	.009	−0.993	1.30 (0.66–2.57)	.45	0.264
	Ovarian cysts (benign) ever diagnosed∗	2.74 (1.51–4.95)	.001	1.007	2.92 (1.35–6.30)	.006	1.072
	Scoliosis ever diagnosed∗	10.1 (2.14–47.2)	.003	2.308	1.16 (0.33–4.08)	.82	0.148
	Asthma ever diagnosed∗	0.31 (0.10–0.94)	.039	−1.186	0.16 (0.03–0.81)	.027	−1.863
	Family history of ovarian cancer in first- or second-degree relative∗	0.28 (0.07–1.06)	.06	−1.290	0.43 (0.03–5.62)	.52	−0.851
	Family history of endometriosis in first- or second-degree relative∗	3.38 (0.69–16.4)	.132	1.218	1.54 (0.39–6.11)	.54	0.432
	Asian or Oriental ethnicity^g^	0.37 (0.17–0.81)	.014	−1.003	1.05 (0.42–2.63)	.92	0.045
	Black ethnicity∗^g^	2.81 (0.91–8.74)	.074	1.034	7.88 (3.03–20.5)	<.001	2.064
	Other/Mixed ethnicity^g^	0.53 (0.24–1.14)	.10	−0.631	1.49 (0.56–3.93)	.42	0.396
	Duration of smoking (years)^a^	0.93 (0.89–0.97)	<.001	−0.073	1.07 (1.02–1.13)	.011	0.071
	Phase I: Nagelkerke's *R*^2^ = 0.54; Hosmer-Lemeshow test = 0.16. Phase II: Nagelkerke's *R*^2^ = 0.54; Hosmer-Lemeshow test = 0.84						

∗ Variables retained in the reduced models. Variables in the best-fitting final models that had opposite directions of effect in phase I and II data sets were dropped in the reduced models. IBS = irritable bowel syndrome.

a Included as a linear term: OR represents unit change.

b Included as a linear term: OR represents unit change from light to moderate to heavy.

c Included as a linear term: OR represents unit change from no pain to 0–3 months, 4–6 months, 7–12 months, 1–5 years, to 5 years ago.

d Included as a linear term: OR represents unit change from 0 to 10.

e Included as a linear term: OR represents unit change from 0, 1, 2, to 3+.

f Included as a linear term: OR represents unit change from none, 1–10, 11–50, to 50+.

g Ethnicity is included as a categorical term, with largest group (white) as reference.

**Table 1:** Association results and regression coefficients from best fitting final full models for stage III and IV endometriosis excluding (model 5) and including (model 7) ultrasound evidence in phase I, and when subsequently applied to phase II.

Model	Variable	Phase I			Phase II		
		OR (95% CI)	*P* value	β	OR (95% CI)	*P* value	β
Stage III and IV no ultrasound (model 5)	Surgery/consultation for ovarian cyst∗	2.61 (1.35–5.06)	.004	0.961	2.65 (1.44–4.86)	.002	0.973
	Surgery/consultation for infertility∗	0.69 (0.39–1.22)	.20	−0.368	0.66 (0.37–1.19)	.16	−0.418
	Menstrual flow^a^	1.45 (0.94–2.24)	.09	0.372	1.00 (0.68–1.47)	.99	−0.002
	Average cycle length∗^b^	0.74 (0.57–0.95)	.019	−0.308	0.77 (0.59–1.01)	.057	−0.257
	Pelvic pain during periods	4.54 (1.60–12.9)	.005	1.513	0.60 (0.23–1.52)	.28	−0.515
	Pain on opening bowels during periods∗	3.61 (1.85–7.04)	<.001	1.283	3.09 (1.39–6.87)	.006	1.129
	Frequency of period pain^c^	0.68 (0.46–0.99)	.043	−0.393	1.04 (0.72–1.51)	.84	0.040
	Period pain limited work/daily activities∗^c^	1.51 (1.11–2.06)	.009	0.412	1.54 (1.11–2.15)	.011	0.434
	Period pain better after bowel movement∗^d^	1.32 (1.00–1.74)	.052	0.276	1.04 (0.77–1.41)	.78	0.043
	Stools looser with period pain∗^d^	0.83 (0.64–1.08)	.17	−0.183	0.80 (0.60–1.07)	.13	−0.222
	Worst severity of pelvic pain at last pelvic exam∗^g^	1.16 (1.02–1.32)	.022	0.151	1.14 (0.99–1.32)	.06	0.135
	Loose stools in general^e^	1.40 (0.92–2.12)	.12	0.334	0.96 (0.64–1.44)	.83	−0.044
	Pelvic pain after urination in general∗^f^	0.67 (0.42–1.05)	.08	−0.404	0.86 (0.56–1.34)	.51	−0.147
	Frequency of nocturia in general∗^g^	0.59 (0.42–0.83)	.002	−0.528	0.85 (0.63–1.15)	.29	−0.161
	Number of live births∗^h^	0.59 (0.40–0.89)	.011	−0.522	0.65 (0.44–0.96)	.028	−0.429
	Ever diagnosed blocked tubes as reason for subfertility∗	0.38 (0.16–0.91)	.029	−0.966	0.64 (0.31–1.29)	.21	−0.453
	Ovarian cysts (benign) ever diagnosed∗	2.35 (1.19–4.62)	.014	0.853	3.81 (2.08–6.97)	<.001	1.336
	Polycystic ovarian syndrome ever diagnosed∗	0.34 (0.10–1.14)	.08	−1.071	0.56 (0.18–1.79)	.33	−0.577
	Family history of asthma in first- or second-degree relative∗	0.40 (0.17–0.93)	.032	−0.924	0.86 (0.39–1.92)	.71	−0.150
	Family history of prostate cancer in first- or second-degree relative	0.23 (0.06–0.84)	.027	−1.466	1.16 (0.34–3.95)	.81	0.150
	Asian or Oriental ethnicity∗^i^	2.73 (1.38–5.41)	.004	1.005	2.83 (1.39–5.74)	.004	1.039
	Black ethnicity∗^i^	3.71 (1.47–9.38)	.006	1.312	4.23 (1.83–9.78)	.001	1.441
	Other/mixed ethnicity^i^	0.90 (0.42–1.93)	.79	−0.104	1.25 (0.55–2.84)	.59	0.224
	Phase I: Nagelkerke's *R*^2^ = 0.47; Hosmer-Lemeshow test = 0.25. Phase II: Nagelkerke's *R*^2^ = 0.42; Hosmer-Lemeshow test = 0.94.						
Stage III and IV ultrasound (model 7)	Ultrasound evidence of cyst/nodule	15.4 (8.2–29.0)	<.001	2.736	11.2 (6.23–20.1)	<.001	2.415
	Average cycle length∗^b^	0.63 (0.47–0.83)	.001	−0.465	0.79 (0.60–1.06)	.11	−0.232
	Pelvic pain during periods∗	1.80 (0.94–3.46)	.076	0.589	1.41 (0.80–2.46)	.23	0.341
	Pain on opening bowels during periods∗	2.58 (1.27–5.26)	.009	0.949	3.38 (1.53–7.46)	.003	1.217
	Pain usually approximately same time in cycle (just after periods)∗	2.06 (0.74–5.78)	.17	0.725	1.95 (0.63–6.04)	.25	0.667
	IBS (Rome III)∗	3.43 (1.24–9.44)	.017	1.231	2.43 (0.76–7.81)	.14	0.887
	IBS-M (Rome III)∗	0.43 (0.13–1.40)	.16	−0.843	0.47 (0.13–1.76)	.26	−0.752
	Frequency of nocturia in general∗^g^	0.71 (0.53–0.94)	.015	−0.350	0.81 (0.64–1.03)	.079	−0.210
	Number of live births∗^h^	0.59 (0.38–0.92)	.019	−0.530	0.85 (0.59–1.24)	.41	−0.159
	Ever diagnosed blocked tubes as reason for subfertility∗	0.28 (0.12–0.67)	.004	−1.270	0.74 (0.38–1.47)	.40	−0.296
	Ovarian cysts (benign) ever diagnosed∗	2.82 (1.56–5.09)	.001	1.037	3.96 (2.24–7.00)	<.001	1.376
	Polycystic Ovarian Syndrome ever diagnosed∗	0.39 (0.10–1.49)	.17	−0.941	0.54 (0.17–1.71)	.29	−0.620
	Asthma ever diagnosed	0.09 (0.02–0.44)	.003	−2.442	1.35 (0.32–5.74)	.69	0.299
	Any atopic condition ever diagnosed	1.64 (0.85–3.17)	.14	0.496	0.53 (0.29–0.96)	.037	−0.640
	Family history of prostate cancer in first- or second-degree relative∗	0.16 (0.04–0.75)	.019	−1.810	0.46 (0.12–1.75)	.26	−0.774
	Asian or Oriental ethnicity∗^i^	4.29 (2.00–9.21)	<.001	1.456	2.05 (1.00–4.21)	.049	0.720
	Black ethnicity∗^i^	5.13 (1.78–14.8)	.002	1.635	7.09 (3.12–16.1)	<.001	1.959
	Other/mixed ethnicity^i^	0.93 (0.41–2.10)	.86	−0.075	1.38 (0.58–3.30)	.47	0.325
	Phase I: Nagelkerke's *R*^2^ = 0.57; Hosmer-Lemeshow test = 0.77. Phase II: Nagelkerke's *R*^2^ = 0.51; Hosmer-Lemeshow test = 0.26						

∗ Variables retained in the reduced models. Variables in the best-fitting final models that had opposite directions of effect in phase I and II data sets were dropped in the reduced models. IBS = irritable bowel syndrome.

a Included as a linear term: OR represents unit change from light to moderate to heavy.

b Included as a linear term: OR represents unit change from <21 days, 22–24 days, 25–28 days, 29–32 days, 33–35 days, to 36+ days.

c Included as a linear term: OR represents unit change from never, occasionally (1 every 3), often (2 every 3), to always.

d Included as a linear term: OR represents unit change from never/rarely, sometimes, often, most of the time, to always.

e Included as a linear term: OR represents unit change from 0 to 10.

f Included as a linear term: OR represents unit change from not at all, less than 1 time in 5, less than half the time, about half the time, more than half the time, to almost always.

g Included as a linear term: OR represents unit change from none, 1 time, 2 times, 3 times, 4 times, to 5+ times.

h Included as a linear term: OR represents unit change from 0, 1, 2, to 3+.

i Ethnicity is included as a categorical term, with largest group (white) as reference.

**Table 2:** Diagnostic value of the final full models for prediction of any-stage and stage III and IV endometriosis in the phase I study and phase II validation populations.

Model parameter	Any-stage endometriosis						Stage III and IV endometriosis					
	Any-stage no ultrasound (model 1)		Any-stage ultrasound (model 3)		Ultrasound scan only		Stage III and IV no ultrasound (model 5)		Stage III and IV ultrasound (model 7)		Ultrasound scan only	
	Phase I	Phase II	Phase I	Phase II	Phase I	Phase II	Phase I	Phase II	Phase I	Phase II	Phase I	Phase II
Area under ROC curve (95% CI)^a^	84.2 (81.1–87.0)	68.3 (63.9–72.4)	87.3 (84.2–90.0)	80.0 (75.6–83.3)	52.0 (48.4–55.5)	51.8 (47.8–55.8)	87.3 (84.3–89.8)	83.3 (79.6–86.6)	90.8 (88.1–93.0)	84.9 (81.4–88.0)	56.4 (52.8–59.9)	53.9 (49.9–57.9)
Sensitivity (%) (95% CI)	74.0 (68.5–79.0)	84.8 (80.1–88.7)	74.3 (68.5–79.6)	58.1 (52.1–63.9)	86.7 (82.7–90.0)	93.7 (90.7–96.0)	85.11 (78.1–90.5)	70.9 (63.5–77.5)	76.71 (69.0–83.3)	82.26 (76.0–87.5)	94.4 (90.0–97.3)	97.4 (94.4–99.0)
Specificity (%) (95% CI)	79.5 (74.8–83.6)	43.5 (36.1–51.1)	84.2 (79.5–88.1)	89.2 (83.5–93.5)	17.3 (13.7–21.3)	9.96 (6.6–14.3)	72.33 (68.0–76.4)	84.70 (79.9–88.7)	89.93 (86.6–92.6)	75.76 (70.5–80.5)	18.4 (15.3–21.7)	10.5 (7.6–14.0)
Optimal cut-off point	0.471	0.302	0.473	0.795	0.479	0.592	0.172	0.452	0.373	0.241	0.262	0.389
Correctly classified (%)	76.4	73.1	79.8	78.7	49.7	58.7	85.5	78.9	87.6	79.3	36.2	42.6
LR+	3.61	1.5	4.7	5.39	1.05	1.04	3.08	4.63	7.62	3.39	1.16	1.09
LR−	0.33	0.35	0.31	0.47	0.77	0.63	0.21	0.34	0.26	0.23	0.30	0.25

*Note:* LR = logistic regression.

a Binomial exact.

## Members of the WERF WHSS Consortium

Hany Abdel-Aleem (Assiut University Hospital, Assiut, Egypt); Mauricio Abrão (University of São Paulo, São Paulo, Brazil); David Adamson (Fertility Physicians of Northern California, Palo Alto, California, USA); Francisco Carmona (University of Barcelona, Barcelona, Spain); Thomas d'Hooghe (University of Leuven, Leuven, Belgium); Carlo de Cicco Nardone (Università Cattolica del Sacro Cuore, Rome, Italy); Fiorenzo de Cicco Nardone (Università Cattolica del Sacro Cuore, Rome, Italy); Bukola Fawole (University of Ibadan, Ibadan, Nigeria); Linda Giudice (University of California, San Francisco, California); Mark Hornstein (Brigham and Women's Hospital, Boston, Massachusetts); Stephen Kennedy (University of Oxford, Oxford, UK); Xishi Liu (Shanghai Obstetrics and Gynaecology Hospital, Shanghai, China); Luis Lombardi (CRC Guatemala City, Guatemala); Xu Min (Guangdong Provincial Hospital of Traditional Chinese Medicine, Guangzhou, China); Stacey Missmer (Brigham and Women's Hospital, Boston, Massachusetts); Felice Petraglia (University of Siena, Siena, Italy); Carlos Petta (State University of Campinas, Campinas, Brazil); Kanok Seejorn (Khon Kaen University, Khon Kaen, Thailand); Pamela Stratton (National Institutes of Health, Washington, DC); Carlos Sueldo (Center for Gynecology and Reproduction, Buenos Aires, Argentina); and Mary Wingfield (National Maternity Hospital, Dublin, Ireland).

## References

[1] Kennedy S., Bergqvist A., Chapron C., D'Hooghe T., Dunselman G., Greb R. (2005). ESHRE guideline for the diagnosis and treatment of endometriosis. Hum Reprod.

[2] Hadfield R., Mardon H., Barlow D., Kennedy S. (1996). Delay in the diagnosis of endometriosis: a survey of women from the USA and the UK. Hum Reprod.

[3] Husby G.K., Haugen R.S., Moen M.H. (2003). Diagnostic delay in women with pain and endometriosis. Acta Obstet Gynecol Scand.

[4] Ballard K., Lowton K., Wright J. (2006). What's the delay? A qualitative study of women's experiences of reaching a diagnosis of endometriosis. Fertil Steril.

[5] Nnoaham K., Hummelshoj L., Webster P., D'Hooghe T., De Cicco Nardone F., De Cicco Nardone (2011). Impact of endometriosis on quality of life and work productivity: a multi-centre study across 10 countries. Fertil Steril.

[6] Vercellini P., Somigliana E., Viganò P., Abbiati A., Barbara G., Crosignani P.G. (2009). Surgery for endometriosis-associated infertility: a pragmatic approach. Hum Reprod.

[7] Chapron C., Fauconnier A., Goffinet F., Bréart G., Dubuisson J.B. (2002). Laparoscopic surgery is not inherently dangerous for patients presenting with benign gynaecologic pathology. Results of a meta-analysis. Hum Reprod.

[8] Hoogeveen M., Dörr P.J., Puylaert J.B.C.M. (2003). Endometriosis of the rectovaginal septum: endovaginal US and MRI findings in two cases. Abdom Imaging.

[9] Rogers P.A.W., D'Hooghe T.M., Fazleabas A., Gargett C.E., Giudice L.C., Montgomery G.W. (2009). Priorities for endometriosis research: recommendations from an International consensus workshop. Reprod Sci.

[10] May K.E., Villar J., Kirtley S., Kennedy S.H., Becker C.M. (2011). Endometrial alterations in endometriosis: a systematic review of putative biomarkers. Hum Reprod Update.

[11] May K.E., Conduit-Hulbert S.A., Villar J., Kirtley S., Kennedy S.H., Becker C.M. (2010). Peripheral biomarkers of endometriosis: a systematic review. Hum Reprod Update.

[12] Eskenazi B., Warner M., Bonsignore L., Olive D., Samuels S., Vercellini P. (2001). Validation study of nonsurgical diagnosis of endometriosis. Fertil Steril.

[13] Austin P., Lee D. (2011). Boosted classification trees result in minor to modest improvement in the accuracy in classifying cardiovascular outcomes compared to conventional classification trees. Am J Cardiovasc Dis.

[14] Chapron C., Barakat H., Fritel X., Dubuisson J.B., Bréart G., Fauconnier A. (2005). Presurgical diagnosis of posterior deep infiltrating endometriosis based on a standardized questionnaire. Hum Reprod.

[15] Fedele L., Bianchi S., Carmignani L., Berlanda N., Fontana E., Frontino G. (2007). Evaluation of a new questionnaire for the presurgical diagnosis of bladder endometriosis. Hum Reprod.

[16] Bleeker S.E., Moll H.A., Steyerberg E.W., Donders A.R.T., Derksen-Lubsen G., Grobbee D.E. (2003). External validation is necessary in prediction research: a clinical example. J Clin Epidemiol.

[17] Simon R., Radmacher M.D., Dobbin K., McShane L.M. (2003). Pitfalls in the use of DNA microarray data for diagnostic and prognostic classification. J Natl Cancer Inst.

[18] Ntzani E.E., Ioannidis J.P.A. (2003). Predictive ability of DNA microarrays for cancer outcomes and correlates: an empirical assessment. Lancet.

[19] Nnoaham K., Sivananthan S., Hummelshoj L., Jenkinson C., Webster P., Kennedy S. (2009). Multi-center studies of the global impact of endometriosis and the predictive value of associated symptoms. J Endometriosis.

[20] Downie W.W., Leatham P.A., Rhind V.M., Wright V., Branco J.A., Anderson J.A. (1978). Studies with pain rating scales. Ann Rheum Dis.

[21] Gwee K.A. (2007). Irritable bowel syndrome and the Rome III criteria: for better or for worse?. Eur J Gastroenterol Hepatol.

[22] Zondervan K.T., Yudkin P.L., Vessey M.P., Jenkinson C.P., Dawes M.G., Barlow D.H. (2001). Chronic pelvic pain in the community—symptoms, investigations, and diagnoses. Am J Obstet Gynecol.

[23] Zondervan K.T., Yudkin P.L., Vessey M.P., Jenkinson C.P., Dawes M.G., Barlow D.H. (2001). The community prevalence of chronic pelvic pain in women and associated illness behaviour. Br J Gen Pract.

[24] (1985). Revised American Fertility Society classification of endometriosis: 1985. Fertil Steril.

[25] Harrell F.E., Lee K.L., Mark D.B. (1996). Multivariable prognostic models: issues in developing models, evaluating assumptions and adequacy, and measuring and reducing errors. Stat Med.

[26] Hosmer D., Lemeshow S. (2000). Applied logistic regression.

[27] Seracchioli R., Mabrouk M., Guerrini M., Manuzzi L., Savelli L., Frascà C. (2008). Dyschezia and posterior deep infiltrating endometriosis: analysis of 360 cases. J Minim Invasive Gynecol.

[28] Jacoby V.L., Fujimoto V.Y., Giudice L.C., Kuppermann M., Washington A.E. (2010). Racial and ethnic disparities in benign gynecologic conditions and associated surgeries. Am J Obstet Gynecol.

[29] Mol B.W., Bayram N., Lijmer J.G., Wiegerinck M.A., Bongers M.Y., van der Veen F. (1998). The performance of CA-125 measurement in the detection of endometriosis: a meta-analysis. Fertil Steril.

[30] Koninckx P.R., Oosterlynck D., D'Hooghe T., Meuleman C. (1994). Deeply infiltrating endometriosis is a disease whereas mild endometriosis could be considered a non-disease. Ann N Y Acad Sci.

[31] Koninckx P.R. (1994). Is mild endometriosis a condition occurring intermittently in all women?. Hum Reprod.

[32] Brosens I.A. (1994). Is mild endometriosis a progressive disease?. Hum Reprod.

[33] Painter J.N., Anderson C.A., Nyholt D.R., Macgregor S., Lin J., Lee S.H. (2011). Genome-wide association study identifies a locus at 7p15.2 associated with endometriosis. Nat Genet.

[34] Calhaz-Jorge C., Mol B.W., Nunes J., Costa A.P. (2004). Clinical predictive factors for endometriosis in a Portuguese infertile population. Hum Reprod.

[35] Moore J., Copley S., Morris J., Lindsell D., Golding S., Kennedy S. (2002). A systematic review of the accuracy of ultrasound in the diagnosis of endometriosis. Ultrasound Obstet Gynecol.

[36] Hudelist G., English J., Thomas A.E., Tinelli A., Singer C.F., Keckstein J. (2011). Diagnostic accuracy of transvaginal ultrasound for non-invasive diagnosis of bowel endometriosis: systematic review and meta-analysis. Ultrasound Obstet Gynecol.

[37] Somigliana E., Vercellini P., Vigano' P., Benaglia L., Crosignani P.G., Fedele L. (2010). Non-invasive diagnosis of endometriosis: the goal or own goal?. Hum Reprod.

[38] McGeechan K., Macaskill P., Irwig L., Liew G., Wong T.Y. (2008). Assessing new biomarkers and predictive models for use in clinical practice: a clinician's guide. Arch Intern Med.

